# Effects of qigong and resistance exercise on improving balance and cognitive ability of community-dwelling older adults

**DOI:** 10.3389/fpubh.2026.1876625

**Published:** 2026-08-20

**Authors:** Wen Fu, Li Zhang, Yu Liao, Qi Zheng, Xiaolei Peng, Jiayu Zou, Tingjin Duan, Xiaochun Chen

**Affiliations:** 1 School of Physical Education and Sport Science, Fujian Normal University, Fuzhou, China; 2 Department of Physical Education and Research, Xiamen Medical College, Xiamen, China; 3 School of General Education, Sichuan Aerospace Vocational College, Deyang, China; 4 Sports Department, Guangdong Nanfang Institute of Technology, Jiangmen, China; 5 Sports Department, Anhui University of Chinese Medicine, Hefei, Anhui, China; 6 General Education College, Fujian Polytechnic of Information Technology, Fuzhou, China; 7 Sports Department, Fujian University of Traditional Chinese Medicine, Fuzhou, China; 8 Department of Physical Education and Research, Fuzhou University Zhicheng College, Fuzhou, China

**Keywords:** Baduanjin, falls, mild cognitive impairment (MCI), older adults, resistance band

## Abstract

**Background:**

To address the challenges posed by an aging society and reduce the incidence of falls and cognitive decline, there is a need for scientifically validated training methods that enhance both balance and cognitive function in older adults.

**Objective:**

To evaluate and compare the effects of Baduanjin and Resistance Band training on improving balance and cognitive function in community-dwelling older adults.

**Methods:**

A total of 93 community-dwelling older adults (mean age: 64.86 ± 3.13 years) were assigned according to the principle of residential proximity into three groups: Baduanjin exercise group (*n* = 32), Resistance Band exercise group (*n* = 30), and control group (*n* = 31). Both intervention groups underwent 16 weeks of training (three sessions per week), with Heart rate (HR) and rating of perceived exertion (RPE) monitored regularly during training sessions to evaluate exercise load. The control group maintained usual daily activities, with step counts recorded three times per week to monitor daily activity levels. Primary outcome measures included the Closed-eyes Single-leg Standing Test (CSST), Timed Up and Go Test (TUG), body mass index (BMI), blood pressure, flexibility (Sit-and- Reach Test and Back Scratch Test), and Montreal Cognitive Assessment (MoCA) scores. The secondary outcome was the correlation between balance ability and cognitive function.

**Results:**

Both intervention groups significantly improved balance and cognitive function compared with the control group (*p* < 0.01), with no significant between-group differences in static balance (CSST), dynamic balance (TUG), or global cognitive function (MoCA total score). However, domain-specific differences emerged: Baduanjin demonstrated significantly greater improvements in flexibility (*p* < 0.05) and systolic blood pressure (*p* < 0.001), as well as in specific MoCA subdomains including executive function and naming ability (*p* < 0.05). The 16-week program achieved 100% attendance with no dropouts. Additionally, dynamic balance (TUG) was significantly correlated with global cognitive function (r = −0.8, *p* < 0.01).

**Conclusion:**

Both Baduanjin exercises and Resistance Band training are effective and feasible interventions for improving balance and cognitive function in community-dwelling older adults, with each modality offering distinct benefits across different outcome domains. These findings support the use of both exercises as safe, accessible options for community-based health promotion in aging populations.

## 1 Introduction

With the accelerated pace of global aging, falls and cognitive decline among older adults have emerged as increasingly serious public health concerns. Statistics indicate that approximately one-third of individuals aged 65 and above experience falls each year (1), and fall-related injury rates significantly increase with age (2). Falls not only diminish quality of life for older adults and increase healthcare burdens but can also lead to severe consequences, including death (3). Furthermore, cognitive impairment is a major risk factor for falls in older adults (4). Therefore, improving balance and cognitive function in older adults is essential for fall prevention and for enhancing their overall quality of life (5, 46).

The relationship between cognition and balance is complex and bidirectional, while the strength of this association appears to be domain-specific and task-specific. Recent systematic reviews demonstrated that executive function exhibits the strongest correlation with balance among all cognitive domains, and that dynamic balance demands greater cognitive resources than static balance (51, 52). Neuroimaging evidence further reveals that older adults exhibit prefrontal-motor cortex overactivation during dual-task standing, reflecting a loss of postural automaticity that links cognitive control with balance performance (6). These findings suggest that balance performance may serve as a behavioral biomarker for cognitive decline and underscore the importance of interventions that simultaneously target both balance and cognitive function in older adults.

Baduanjin is a traditional Chinese mind–body exercise consisting of eight gentle movements synchronized with deep breathing and mental focus. With a simpler structure and lower motor skill threshold than practices such as Tai Chi, it is particularly accessible for older adults (7). Through slow shifts in the center of gravity, extensive limb stretches, and controlled breathing, Baduanjin enhances lower-limb flexibility and balance control, while also reducing fall risk and improving fall-related motor control (8–11, 50). Beyond its physical benefits, Baduanjin enhances cognitive function through multiple neurobiological and psychological pathways. First, the attentional demands of coordinating movement, breathing, and mental focus engage executive control networks and may promote neuroplastic changes in prefrontal and hippocampal regions (12, 54). Second, the meditative component of Baduanjin may improve cerebral perfusion and increase levels of brain-derived neurotrophic factor (BDNF), a key regulator of synaptic plasticity and neuronal survival (13). Empirical evidence confirms that Baduanjin practice significantly increases peripheral blood BDNF levels in older adults, and this increase correlates positively with cognitive improvements (13). Third, the simultaneous processing of motor and cognitive information during practice constitutes a form of dual-task training, which has been shown to enhance cognitive reserve and executive function (14, 55). Consistent with these mechanistic propositions, multiple meta-analyses have confirmed that Baduanjin significantly improves global cognition, memory, and executive function in older adults, including those with mild cognitive impairment, with no adverse effects reported [Yao et al., 2023; (7, 12, 15)]. Improvements have been observed across multiple domains, including short-term memory, verbal fluency, and Stroop task performance. However, the existing evidence is limited by substantial heterogeneity and a scarcity of high-quality studies. Nevertheless, Baduanjin emerges as a safe, low-cost, and accessible non-pharmacological intervention for promoting both balance and cognitive health in aging populations.

However, despite its promising potential, existing studies on Baduanjin have several limitations. First, the generalizability of current findings is constrained by relatively short intervention durations (typically less than 12 weeks), which limits the evaluation of long-term effects and the sustainability of benefits (8, 16). Second, many studies have not rigorously monitored exercise load and daily activity levels, potentially compromising the accuracy of their results (16). Notably, while evidence suggests that significant balance gains begin to emerge with a minimum of 12 weeks of practice (with 30–49 min per session, 5–7 times per week identified as the optimal dosage) (10), the optimal parameters for cognitive outcomes remain unclear. Third, and most critically for the present study, most research either lacks an active control group that engages in exercise or only employs low-intensity aerobic exercise (such as walking) as a control (17). While some studies have compared Tai Chi or mind–body exercises with walking or general exercise, such controls have limited effects on muscle strength and balance, making it difficult to distinguish whether cognitive improvements arise from the specific mind–body characteristics of Baduanjin or from general physical activity (18).

To address this interpretive gap, an active control is required that can separate two potential pathways through which exercise might improve cognition. One pathway is central neurocognitive engagement: Baduanjin demands attentional regulation, motor coordination, and dual-task processing, all of which may directly enhance executive function. This possibility is supported by evidence that mind–body exercise confers cognitive benefits exceeding those of purely aerobic or resistance training, and that interventions explicitly incorporating dual-task elements produce the largest gains in executive function among older adults with cognitive impairment (53, 55). The other pathway is peripheral physiological adaptation: any form of physical exertion may improve cerebral perfusion and upregulate BDNF, regardless of cognitive engagement during exercise. However, disentangling these pathways requires a comparator that can produce physical fitness improvements comparable to Baduanjin while minimizing its cognitive demands.

Resistance band training offers a comparator. This modality primarily imposes external mechanical loads on peripheral muscles through relatively simple, repetitive movements, yet randomized controlled trial evidence demonstrates that it produces improvements in physical fitness, including handgrip strength, gait speed, and short physical performance battery scores, that are comparable to those of Baduanjin (55). Notably, resistance band training has also been shown to improve working memory performance in older adults, with functional near-infrared spectroscopy revealing that such improvements are accompanied by optimized prefrontal cortex activation patterns under increasing cognitive loads (19). This suggests that resistance band training can yield cognitive benefits, but likely through peripheral physiological pathways rather than central cognitive engagement during the exercise itself.

Despite the respective evidence supporting both modalities, a critical knowledge gap persists: no study has directly compared these two mechanistically distinct exercise interventions in a head-to-head trial among community-dwelling older adults. Because they differ substantially in cognitive engagement during practice yet both produce peripheral physiological adaptations, it remains unclear whether the cognitive benefits conferred by Baduanjin are specific to its mind–body characteristics or shared across exercise modalities that improve physical fitness. To address this gap, the present study was designed to answer two specific questions: (1) Do Baduanjin and resistance band training differentially affect balance and cognitive function over a 16-week period? (2) To what extent are improvements in balance correlated with improvements in cognitive performance, and how are these relationships moderated by exercise type? Accordingly, a 16-week non-randomized controlled trial was conducted to compare the impacts of Baduanjin and Resistance Band training on balance, cognitive function, and physical fitness in community-dwelling older adults. This study hypothesized that both interventions would significantly improve balance and cognitive function relative to baseline, but did not presuppose the superiority of either modality. Given their distinct movement characteristics (Baduanjin emphasizing slow, coordinated movements with mental focus, and resistance band training focusing on external mechanical loading through repetitive movements), potential differences in efficacy profiles across outcome domains were anticipated. A further hypothesis was that improvements in balance would correlate significantly with improvements in cognitive performance, and that this correlation might be moderated by exercise type.

## 2 Materials and methods

### 2.1 Sample size

This study used G*Power 3.1 to estimate the required sample size. The effect size (f) was set at 0.25 (considered a small effect), and the power value (power: l—βerr prob) was set at 0.80. The experiment involved three groups, and data were collected pre- and post- intervention. The required sample size was ultimately determined to be 42 participants. Considering an anticipated dropout rate of 20%, at least 51 participants were recruited. The expected effect size of each primary outcome indicator was clearly defined before sample size calculation. Post-hoc statistical power analysis was conducted after data collection to verify the statistical efficacy of the final sample size of 93 participants.

### 2.2 Participants

In Xiangzhou District of Zhuhai City, Guangdong Province, China, a total of 121 older adults aged 60–70 years were recruited. This study recruited subjects from the associations for older adults under the government and large mature communities, and the intervention site was set in the community activity center, a daily activity place for older adults with high participation convenience. The entire recruitment and intervention process relied on the organization and management of communities and associations, which had strong organizational constraints and a sense of belonging; at the same time, the research team provided continuous companionship and on-site guidance to improve the compliance of older adults. These multiple factors jointly ensured that all 93 subjects participated in the whole process without any loss to follow-up, and the sample size was much higher than the 42 people calculated by G*Power, resulting in more sufficient statistical test power. After screening, 93 subjects met the inclusion criteria and were included in the study (excluded:11 due to scheduling conflicts; 7 with prior Baduanjin experience; and 2 with hearing impairments). The eligible participants were then divided into three groups based on their residential proximity rather than random allocation. This grouping strategy was adopted to recruit a sufficient number of participants, but it may introduce selection bias, sociocultural and environmental confounding factors. The uneven distribution of educational levels across different communities further resulted in significant between-group differences in baseline cognitive indicators. Inclusion Criteria: aged 60–70 years and able to walk and stand independently; no history of Qigong or Resistance Band training (within the past two years); no regular physical exercise for the past six months (defined as at least two sessions per week, each session lasting at least 20 min at a moderate or higher intensity); willing to participate in both the experimental exercises and the indicator assessments, with all participants signing informed consent prior to the experiment. Exclusion Criteria: uncontrolled blood pressure (systolic BP ≥ 160 mmHg, diastolic BP ≥ 100 mmHg, or systolic BP ≤ 90 mmHg); long-term unhealthy behaviors, such as alcohol abuse or smoking; Montreal Cognitive Assessment (MoCA) scores lower than 16 (20); contraindications to exercise, such as musculoskeletal disorders, neurological disorders, respiratory diseases, metabolic or endocrine disorders, or psychiatric conditions. Strict management and multiple guarantee measures were adopted to improve participants’ adherence: all interventions were carried out in community activity centers close to participants’ residences; community staff and research team members provided on-site supervision and accompanying guidance throughout the training; regular communication and health reminders were conducted for all participants. The above measures ensured a 100% attendance rate and zero dropout during the 16-week intervention. The final sample size (*n* = 93) was much higher than the pre-estimated 42 participants, resulting in sufficient statistical test power.

### 2.3 Study design

This study involved two experimental groups—Baduanjin exercises and Resistance Band exercises—and one control group. In the experimental groups, two coaches, each with over five years of experience in their respective disciplines, conducted instruction sessions for Baduanjin and Resistance Band exercises, respectively, at the same time and location. Daily physical activity levels of all participants in both experimental groups and the control group were monitored by recording step counts using the WeChat step counts of smartphones. All participants were required to carry their smartphones during their daily activities to record daily activity data, except during the exercise intervention sessions, in which subjects in the intervention groups were instructed not to carry their phones. It should be noted that this monitoring method has limitations: different smartphone models may lead to minor errors in step counting, and some older adult participants may forget to carry their phones in daily life, which may affect the accuracy of activity data. For future studies, wearable sports bracelets or professional sports watches are recommended to obtain more precise daily activity data. Weekly step count data from all three groups were analyzed to assess differences in daily activity levels, ensuring that the activity levels of all groups remained comparable and thus safeguarding the accuracy of the experimental outcomes.

The study was conducted over a 16-week period, from March 2023 to November 2023, at a Community Activity Center in Xiangzhou District, Zhuhai City, Guangdong Province, China.

One assessment of experimental indicators was conducted before the intervention, and another assessment was performed for all three groups following the 16-week intervention. During the intervention, in each training session at three time points—before, during, and after the session—subjects in the two exercise groups were sampled for heart rate (HR) and the rating of perceived exertion (RPE) data. These two convenient and comparatively accurate methods were employed to monitor exercise load throughout the intervention, thereby ensuring that experiment outcomes could be attributed to differences in exercise modality. Finally, the data were compiled and subjected to comparative analysis.

HR and RPE are two important indicators for assessing exercise load, as they reflect the physiological response and subjective perception related to exercise intensity (21, 22). Exercise load refers to the physiological and psychological stress imposed on the body during a specific exercise, encompassing factors such as intensity, duration, and type of activity. Because HR and RPE are directly linked to physiological mechanisms, they serve as precise tools for accurately reflecting exercise load (23, 24).

HR is an objective physiological indicator that is directly linked to the body’s demand for exertion. During exercise, the increase in heart rate meets the muscles’ needs for oxygen and energy. According to Silva et al. (25), there is a strong linear relationship between heart rate and oxygen consumption, indicating that heart rate can, to some extent, reflect both the intensity and energy expenditure of aerobic exercise. Consequently, heart rate serves as an effective physiological indicator of exercise load, especially in aerobic activities.

RPE is a subjective assessment tool that measures an individual’s perception of exercise load under varying intensities. During high-intensity exercise, a trainee’s subjective perception often intensifies in parallel with physiological stress responses such as increased heart rate and respiratory rate (26). Research has demonstrated that RPE is significantly correlated with physiological indicators—including heart rate and lactic acid accumulation—thus serving as an effective means of gauging exercise intensity and the level of fatigue experienced by the trainee (27).

The primary reason HR and RPE can reflect exercise load is that they provide feedback on the body’s response from both physiological and subjective perspectives. HR, based on objective physiological mechanisms, offers precise information regarding exercise intensity, while RPE captures the individual’s subjective experience of exercise-induced stress. Consequently, combining these two measures enables a comprehensive evaluation of exercise load, which is crucial for optimizing exercise programs and monitoring training outcomes. The overall exercise intensity of both interventions was categorized as low-to-moderate, with mean heart rates of 81–89 beats per minute and RPE scores ranging from 9 to 11. This mild-load design was determined based on multiple rationales. First, we adopted high-posture Baduanjin and low-tension yellow resistance bands in the early training stage to fully guarantee the physical safety of older participants and sustain long-term adherence across the 16-week intervention cycle. Second, the core training targets of this trial were balance control and cognitive function, rather than cardiorespiratory endurance or maximal muscle strength. Both Baduanjin and resistance band protocols rely heavily on repeated weight shifting, multi-directional posture adjustment and dual-task coordination, which effectively stimulate neuromuscular and cognitive integration without requiring high heart rate loads. Even under low-to-moderate intensity, consistent long-term practice can produce measurable improvements in static and dynamic balance (10).

The exercise interventions consisted of the traditional Chinese fitness method Baduanjin, and the Resistance Band exercises, which incorporated eight movements. Baduanjin comprises eight forms that are performed symmetrically on both sides, with four forms targeting the upper limbs and four focusing on the lower limbs. A complete cycle takes 12 min. Participants were instructed to coordinate their breathing with the movements. For example, they were asked to inhale at the start of a movement and exhale at the end, to inhale during the extension phase and exhale during the contraction phase, and to inhale during relaxation and exhale during tightening. The Resistance Band exercises involved movements similar to those in Baduanjin, with four movements each for the upper and lower limb muscle groups. During the first month, training was conducted using a yellow resistance band; from the second month until the end of the experiment, a green resistance band was used. The resistance progressively increased from 5 to 18 pounds. Training sessions were scheduled three times per week, with each session lasting 90 min (from 7:00 to 8:30 a.m.). The entire exercise intervention lasted for 16 weeks.

#### 2.3.1 Baduanjin exercises training group

All training sessions were instructed by certified coaches with professional judging experience in traditional Chinese fitness and resistance training. To keep the intervention conditions consistent, the eight movements of the resistance band training were designed to mimic the movement patterns of Baduanjin. Baduanjin is a traditional Chinese fitness practice that originated in ancient times. It employs gentle, slow, and fluid movements to stretch muscles and tendons and to unblock meridians, thereby harmonizing body functions and improving health (28, 29). Research indicates that several Baduanjin forms that stimulate the leg and core muscle groups (such as “Drawing the Bow to Shoot the Eagle,” “Sway the Head and Shake the Tail,” “Two Hands Hold the Feet,” and “Clench the Fists and Glare Fiercely”) utilize the lower limbs as the primary point of force and shift the center of gravity through compression and stretching of the leg and core muscles. These movements effectively enhance lower-limb and core strength in older adults, particularly strengthening key muscles such as the adductors, quadriceps, and the muscles of the waist and back, which in turn improves balance (30). In addition, Baduanjin training is performed predominantly in a squatting stance, characterized by a bent-knee posture. Repeated alternation between standing upright and squatting engages the muscles around the hips, knees, and ankles in multidirectional movements, thus enhancing lower-limb strength and flexibility, promoting blood circulation in the lower extremity tissues, stimulating the nervous system, and ultimately improving balance (31).

Additionally, the positive effects of Bajuanjin on the nervous system of older adults may also enhance their balance capabilities. The multiple and multi-directional head movements in Baduanjin exercises—including upward tilting, left and right turning, and circular rotations—improve cervical spine flexibility, stimulate blood circulation in the head, and thereby enhance the functions of the cerebellum and vestibular organs. This effectively helps prevent incidents such as falls (32, 33).

In each session of the intervention, the intervention group performed two circuits, each consisting of two 15-min rounds of the eight Baduanjin forms. Each movement was repeated 6 times per round and a 2-min rest between two rounds. Progressive adjustment of exercise load was achieved by modifying the height of the subjects’ center of gravity, in other words, by altering the bending angles between the thigh and calf in the horse stance and bow stance. During the first eight weeks, the prescribed standard was to maintain a bending angle of approximately 135° between the upper and lower legs in both the horse stance and bow stance, whereas in the final eight weeks, the angle was reduced to around 100°. This angle adjustment was uniformly conducted at the 8th week based on the pre-designed training protocol for all participants.

#### 2.3.2 Resistance band exercises training group

Resistance Band exercises are a popular method in modern physical training. Trainees use resistance bands with varying loads (measured in pounds) to perform a variety of resistance exercises, which enhance physical abilities, such as muscle strength, endurance, explosive power, overall flexibility, etc. (34).

In the intervention, the resistance bands used had resistance levels of 5 pounds (yellow) and 18 pounds (green). The eight movements (standing chest press, standing elbow flexion, reverse fly, standing overhead press, standing knee raise, standing hip extension, standing knee flexion, and standing knee extension) targeted the major muscle groups of both the upper limbs (including the pectoralis major, latissimus dorsi, trapezius, etc.) and the lower limbs (including the quadriceps, iliopsoas, gluteus maximus, and hamstrings).

During the intervention, in each session, the experimental group performed two circuits, each consisting of 6 rounds, with a 30-s rest between each movement. Progressive loading was achieved by changing the resistance level of the resistance bands. During the first eight weeks, the participants used a 5-pound resistance band (yellow); during the subsequent eight weeks, they used an 18-pound resistance band (green).

Baduanjin exercises are characterized by frequent weight shifting toward the limit of stability and sustained squatting motions. For the Resistance Band exercise, participants were required to control movement rhythm and coordinate breathing with muscle contractions. For forms 1 and 2, participants performed each movement 6 times. For Baduanjin in Form 1, participants followed instructions accompanied by preset music. For Resistance Band Form 2, participants rested for 1 min after completing each movement.

The replacement of resistance bands was implemented at the 8th week in accordance with the pre-established training schedule to ensure unified progressive loading for all participants. All participants were required to maintain a steady movement speed and coordinated breathing throughout training to guarantee consistent exercise intensity among individuals.

### 2.4 Outcome measures

#### 2.4.1 Main indicators

##### 2.4.1.1 Balance ability

Static Balance: The Closed-eyes Single-leg Standing Test (CSST) is an effective method for assessing static balance, particularly suitable for older adults. Before the test, examiners confirmed that participants had no dizziness or physical fatigue. All tests were performed on anti-slip PVC mats, and participants stood against the wall with examiners standing on both sides for full protection to avoid falls. Participants remove their shoes, socks, and outerwear, and stand on a testing mat. They place their hands on the posterior lateral aspects of their waist, keep both feet together, and face directly forward in an upright posture. Upon receiving the command to start, they immediately close their eyes and, using either the left or right foot as the supporting foot, they lift the opposite leg by flexing it so that the sole is no longer in contact with the ground. Moreover, the raised foot or leg must not come into contact with the supporting limb. Timing begins when the lifted foot leaves the ground and ends when it touches the ground again or when the supporting foot moves (35). Throughout the test, participants keep their eyes closed while an observer stands directly in front of them. The standing duration is recorded in seconds. Both the left and right legs are tested, with each test repeated three times at one-minute intervals between trials, and the best performance from the three trials is taken. Excellent test–retest reliability was observed for the left-leg assessment (ICC [3, 1] = 0.97, 95% CI [0.93, 0.99]), while the right-leg assessment demonstrated good reliability (ICC [3, 1] = 0.77, 95% CI [0.50, 0.90]).

Dynamic balance: the Timed Up and Go Test (TUG) is a reliable and effective method for assessing dynamic walking ability. In this test, subjects begin seated on a chair. Upon receiving the “start” command, the subject stands up, walks three meters at their normal pace, turns around a cone, and then returns along the same path to sit down, which marks the end of the test (36). The test is conducted three times, with a one-minute interval between each trial. Subjects first perform one practice trial to become familiar with the procedure and then complete two formal test trials. Scoring is based on the time (in seconds) taken to finish one cycle of the test, with the final test score being the average of the two formal trials. The TUG demonstrated excellent test–retest reliability (ICC [3, 1] = 0.96, 95% CI [0.90, 0.98]).

Blood pressure (BP): measurements are taken within one hour after waking up in the morning, after urination, and while fasting. If the subject is taking antihypertensive medication, the measurement should be conducted before taking the medication. The subject should fully extend their arm with the cuff positioned at heart level and adjusted to moderate tightness. An automatic reading can be obtained by pressing the power button on the blood pressure monitor. During the measurement, the subject should not speak or move. Two readings are taken, and the average value is used.

Sit-and-Reach Test (SRT): this test is used to assess the range of motion in joints such as the torso, waist, and hips while at rest, effectively reflecting body flexibility. In this test, a participant sits on the floor with both legs fully extended and the feet placed flat on a testing platform—set about 10 to 15 centimeters apart. The participant then bends forward from the waist, extending both arms straight ahead, and gradually pushes a sliding marker forward using the tips of their middle fingers until they can no longer extend further. The inner edge of the foot platform is designated as the zero point; any reach beyond this point is recorded as a positive value, while a reach falling short is recorded as a negative value. The score is measured in centimeters, rounded to one decimal place. The test is performed twice, and the best result is recorded. The SRT demonstrated excellent test–retest reliability (ICC [3, 1] = 1.00, 95% CI [0.99, 1.00]).

Body Mass Index (BMI): in a fasting state upon waking, the subject removes shoes, socks, and outerwear, and stands on the measurement platform of a height and weight testing device (HHTC100/200-ST) manufactured by Huaxia Huihai Technology Co., Ltd—a producer of physical fitness testing instruments that meet Chinese national standard. Two measurements are taken and the average value is recorded.

##### 2.4.1.2 Flexibility

Back Scratch Test (BST): this test is a simple yet accurate method for assessing shoulder flexibility. During the test, the participant stands upright. One hand is positioned behind the same-side shoulder with the forearm rotated forward and the fingers spread, while the other hand is placed on the back with the fingers similarly extended. The participant then attempts to use both hands to touch a measuring ruler. The distance between the hands is recorded—the closer the hands are, the better the flexibility. Scoring: Record the distance between the two hands (or the amount of overlap). If the hands overlap, the value is recorded as positive; if they do not meet, the value is recorded as negative. Measurements are precise to the nearest centimeter, and the best result of two trials is taken as the final test score. Excellent test–retest reliability was observed for both the right-side (ICC [3, 1] = 0.99, 95% CI [0.96, 0.99]) and left-side (ICC [3, 1] = 0.96, 95% CI [0.87, 0.99]) measurements.

Montreal Cognitive Assessment (MoCA): the MoCA is a screening tool used for the rapid detection of cognitive dysfunction in older adults. Since the participants in this study are from China, the Beijing version was utilized. The assessment covers seven general cognitive domains—attention, executive function, memory, language, visuospatial skills, abstract reasoning, calculation, and orientation—measured through a total of eleven items (20). The total score is 30 points: scores of 26 or above are considered normal; scores between 26 and 22 indicate good performance; scores between 22 and 16 suggest mild cognitive impairment; and scores below 16 indicate severe impairment. Moreover, if a participant has 12 or fewer years of education, one point is added to their total score. This education-level correction was strictly implemented in all participants’ final MoCA scores. Based on the baseline data of all participants in this study, the Cronbach’s *α* coefficient of the MoCA was calculated as 0.872, demonstrating excellent internal consistency of this scale in the present sample (47).

Consistent with the study flowchart, all outcome assessments were completed under a single-blind design. After group assignment, the evaluators were only informed of the unified intervention protocols without knowing the specific group division of each participant. Two professional assessors independently completed all tests in a back-to-back manner, while a third researcher was responsible for data sorting, statistical analysis and result interpretation to minimize assessment bias.

This study mainly evaluated balance ability as a surrogate indicator of fall risk, while the actual fall incidence and professional fall risk scores were not measured, which is a limitation of this research.

### 2.5 Statistical analysis

Data were statistically analyzed using SPSS 27.0 (IBM Corp., Armonk, NY). Continuous variables are presented as mean ± standard deviation (M ± SD), while categorical variables are expressed as frequencies (percentages). Prior to the intervention, one-way analysis of variance (One-Way ANOVA) was conducted to compare the three groups (Baduanjin exercise group, Resistance Band exercise group, and control group) in terms of demographics (e.g., age, gender), baseline physical functions (balance and flexibility), and cognitive functions (total score and sub-items of MoCA), thereby verifying baseline homogeneity among the groups. If the data did not meet the assumptions of normality (as determined by the Shapiro–Wilk test) or homogeneity of variance (as assessed by Levene’s test, *p* < 0.05), the non-parametric Kruskal-Wallis test was applied.

Repeated Measures ANOVA (RM ANOVA) was applied to evaluate the intervention’s time effect (pre- vs. post-intervention), between-group effect (differences among the three groups), and time × group interaction effect. The model included group (between-subject factor) and time (within-subject factor), with age and gender entered as covariates to control for potential confounding factors. If the interaction effect was significant (*p* < 0.05), simple effects analyses were conducted to compare differences among groups at various time points. For cognitive function indicators that showed significant baseline differences (e.g., executive function, naming ability), Analysis of Covariance (ANCOVA) was used to adjust the post-intervention data. Baseline values were included as covariates in the model to calculate the means after adjustment and the significance of their differences among the groups. If the ANOVA or ANCOVA results indicated significant main effects, pairwise comparisons were performed using the Bonferroni method to control for Type I error inflation. Effect sizes are expressed as η^2^_p_ (Partial Eta Squared), with η^2^_p_ ≥ 0.01 indicating a small effect, ≥ 0.06 a medium effect, and ≥ 0.14 a large effect (37).

**Figure 1:**
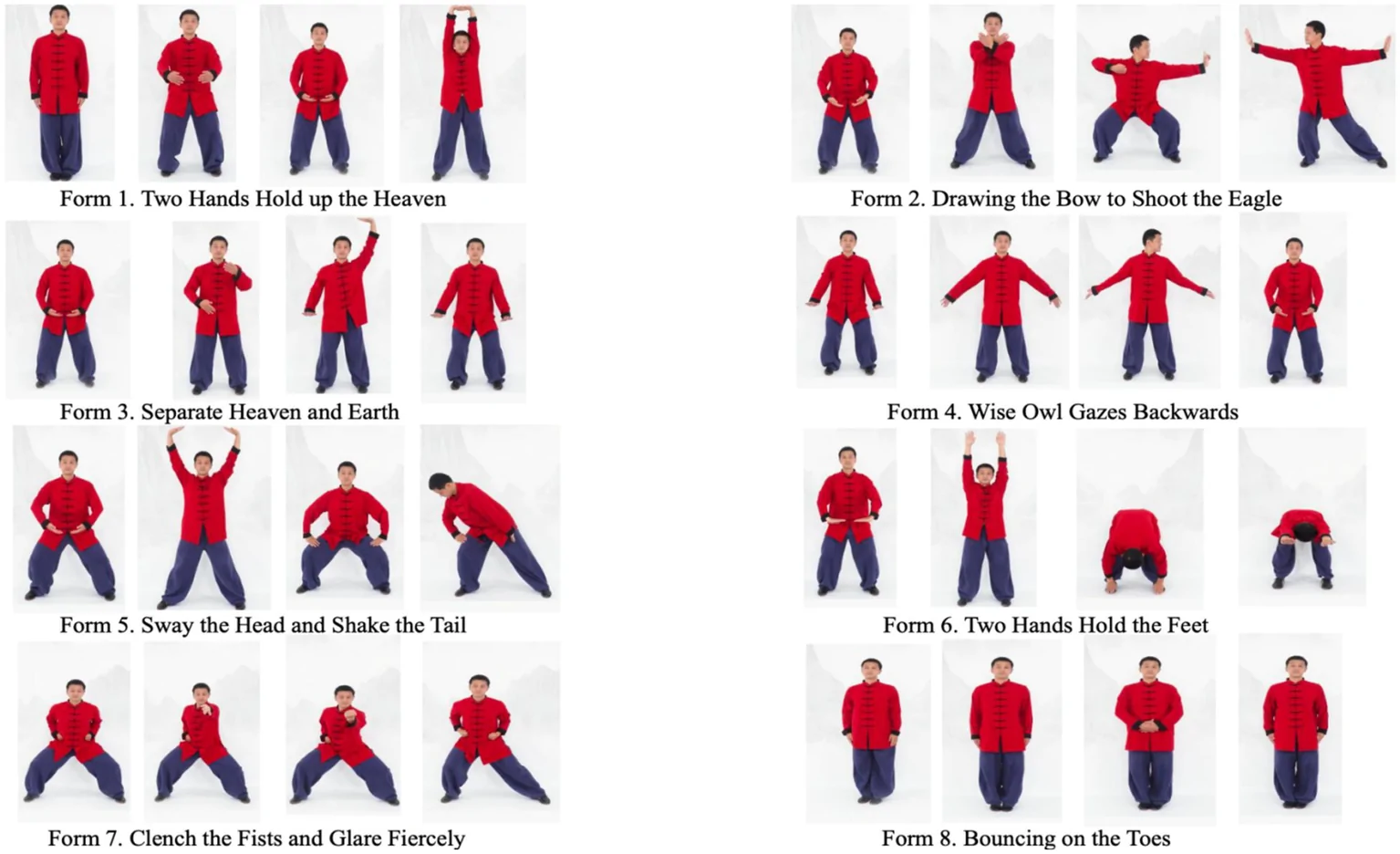
Eight forms of Baduanjin.

Pearson correlation coefficients were used to assess the association between balance ability (static balance measured by the CSST and dynamic balance measured by TUG) and cognitive function (total score and sub-items of MoCA). If the variables did not meet the assumption of normality, Spearman’s rank correlation coefficient is used instead (Figure 1).

## 3 Results

### 3.1 Baseline characteristics and compliance

No significant differences were found among the three groups (Baduanjin exercise group, Resistance Band exercise group, and control group) regarding age, education level, income, or baseline balance function (CSST and TUG) (*p* > 0.05). However, the control group had a significantly higher baseline MoCA total score compared with the intervention groups (*p* < 0.05), so this variable was controlled for as a covariate in subsequent analyses (Table 1). Detailed pairwise comparison results for all baseline indicators across the three groups are presented in Table 2.

**Table 1:** Demographic and the experimental index of participants.

Variable	Category	QG	RE	CON	*p*
Age in years (mean ± SD)		65.30 ± 3.21	65.10 ± 2.75	64.22 ± 3.39	0.351
Education	Primary (n)	11 (34.4%)	10 (33.3%)	12 (38.7%)	1
	Secondary	13 (40.6%)	13 (43.3%)	10 (32.3%)	
	University	8 (25.0%)	7 (23.3%)	9 (29.0%)	
Income	≤1,000 RMB	10 (31.3%)	9 (30%)	12 (38.7%)	0.826
	1,001–3,000 RMB	12 (37.5%)	10 (33.3%)	9 (29.0%)	
	≥3,001 RMB	10 (31.3%)	11 (36.7)	10 (32.3%)	
Live alone	Yes	25 (78.1%)	21 (70.0%)	26 (83.9%)	0.438
	No	7 (21.9%)	9 (30.0%)	5 (16.1%)	
Height (m)		1.62 ± 0.06	1.61 ± 0.04	1.62 ± 0.03	0.989
Weight (kg)		60.44 ± 5.55	60.31 ± 513	60.53 ± 4.35	0.985
BMI		23.19 ± 1.22	23.13 ± 1.55	23.16 ± 1.31	0.993
CSST (s, L)		7.26 ± 3.88	7.04 ± 4.15	7.45 ± 4.21	0.927
CSST (s, R)		5.15 ± 2.61	5.49 ± 3.32	5.45 ± 3.00	0.888
TUG		10.26 ± 1.38	10.23 ± 1.49	10.27 ± 1.55	0.993
2 min Leg lift test		58.63 ± 10.29	61.07 ± 10.20	59.42 ± 9.63	0.625
Visuospatial/Executive		4.50 ± 0.72	4.30 ± 0.70	4.77 ± 7.72	0.037
Nominal function		2.28 ± 0.68	1.87 ± 0.51	2.06 ± 0.57	0.027
Attention span		5.09 ± 0.78	4.97 ± 0.81	5.03 ± 0.66	0.801
Linguistic ability		2.56 ± 0.50	2.67 ± 0.48	2.55 ± 0.51	0.600
Abstract reasoning		1.75 ± 0.44	2.13 ± 0.68	2.16 ± 0.64	0.011
Delayed recall		2.72 ± 1.14	2.40 ± 1.00	2.97 ± 1.14	0.136
Orientation		4.94 ± 0.81	5.07 ± 0.83	5.10 ± 0.75	0.699
Moca score		23.84 ± 1.46	23.40 ± 1.40	24.65 ± 1.68	0.003

RE is the resistance exercise training group; QG is the qigong training group; CON is the control group.

**Table 2:** Baseline multiple comparisons among three groups.

Index	Group	Sample size	Mean	SD	Comparison group	*p*-value
Pre_Height (m)	CON	31	1.615	0.050	RE	0.886
					QG	0.909
	RE	30	1.616	0.033	CON	0.886
					QG	0.975
	QG	32	1.616	0.061	CON	0.909
					RE	0.975
Pre_Wt (kg)	CON	31	60.310	5.130	RE	0.861
					QG	0.918
	RE	30	60.537	4.350	CON	0.861
					QG	0.940
	QG	32	60.441	5.548	CON	0.918
					RE	0.940
Pre_BMI	CON	31	23.131	1.550	RE	0.934
					QG	0.975
	RE	30	23.160	1.305	CON	0.934
					QG	0.909
	QG	32	23.120	1.219	CON	0.975
					RE	0.909
Pre_Back Scratch (cm, L)	CON	31	−1.130	4.056	RE	0.505
					QG	0.708
	RE	30	−1.800	3.438	CON	0.505
					QG	0.764
	QG	32	−1.500	4.189	CON	0.708
					RE	0.764
Pre_Back Scratch (cm, R)	CON	31	−2.100	4.354	RE	0.244
					QG	0.442
	RE	30	−0.930	3.523	CON	0.244
					QG	0.678
	QG	32	−1.340	3.686	CON	0.442
					RE	0.678
Pre_Grip Strength (N, L)	CON	31	21.390	2.481	RE	0.147
					QG	0.722
	RE	30	20.417	2.444	CON	0.147
					QG	0.266
	QG	32	21.156	2.839	CON	0.722
					RE	0.266
Pre_Grip Strength (N, R)	CON	31	20.590	2.775	RE	0.143
					QG	0.164
	RE	30	19.467	3.225	CON	0.143
					QG	0.922
	QG	32	19.541	2.901	CON	0.164
					RE	0.922
Pre_30 s Sit-to-Stand (times)	CON	31	17.130	2.513	RE	0.202
					QG	0.679
	RE	30	16.130	3.256	CON	0.202
					QG	0.379
	QG	32	16.810	3.247	CON	0.679
					RE	0.379
Pre_30 s Arm Curl (times, L)	CON	31	20.550	4.146	RE	0.606
					QG	0.694
	RE	30	20.070	3.787	CON	0.606
					QG	0.896
	QG	32	20.190	2.867	CON	0.694
					RE	0.896
Pre_30 s Arm Curl (times, R)	CON	31	24.230	3.783	RE	0.479
					QG	0.489
	RE	30	23.600	3.654	CON	0.479
					QG	0.977
	QG	32	23.630	2.814	CON	0.489
					RE	0.977
Pre_2 min Step Test (times)	CON	31	59.420	9.626	RE	0.524
					QG	0.754
	RE	30	61.070	10.201	CON	0.524
					QG	0.341
	QG	32	58.630	10.289	CON	0.754
					RE	0.341
Pre_TUG (s)	CON	31	10.274	1.548	RE	0.907
					QG	0.966
	RE	30	10.229	1.493	CON	0.907
					QG	0.940
	QG	32	10.258	1.377	CON	0.966
					RE	0.940
Pre_Sit-and-Reach (cm)	CON	31	6.529	2.783	RE	0.941
					QG	0.557
	RE	30	6.473	2.914	CON	0.941
					QG	0.611
	QG	32	6.091	3.144	CON	0.557
					RE	0.611
Pre_Vital Capacity	CON	31	1948.290	207.751	RE	0.839
					QG	0.690
	RE	30	1958.900	195.949	CON	0.839
					QG	0.549
	QG	32	1927.810	205.251	CON	0.690
					RE	0.549
Pre_CSST (s, L)	CON	31	7.448	4.208	RE	0.698
					QG	0.857
	RE	30	7.041	4.147	CON	0.698
					QG	0.832
	QG	32	7.262	3.878	CON	0.857
					RE	0.832
Pre_CSST (s, R)	CON	31	5.445	2.995	RE	0.956
					QG	0.696
	RE	30	5.488	3.317	CON	0.956
					QG	0.658
	QG	32	5.148	2.609	CON	0.696
					RE	0.658

RE is the resistance exercise training group; QG is the qigong training group; CON is the control group; CSST refers to the Closed-eyes Single-leg Standing Test; TUG is the Timed Up and Go Test.

**Figure 2:**
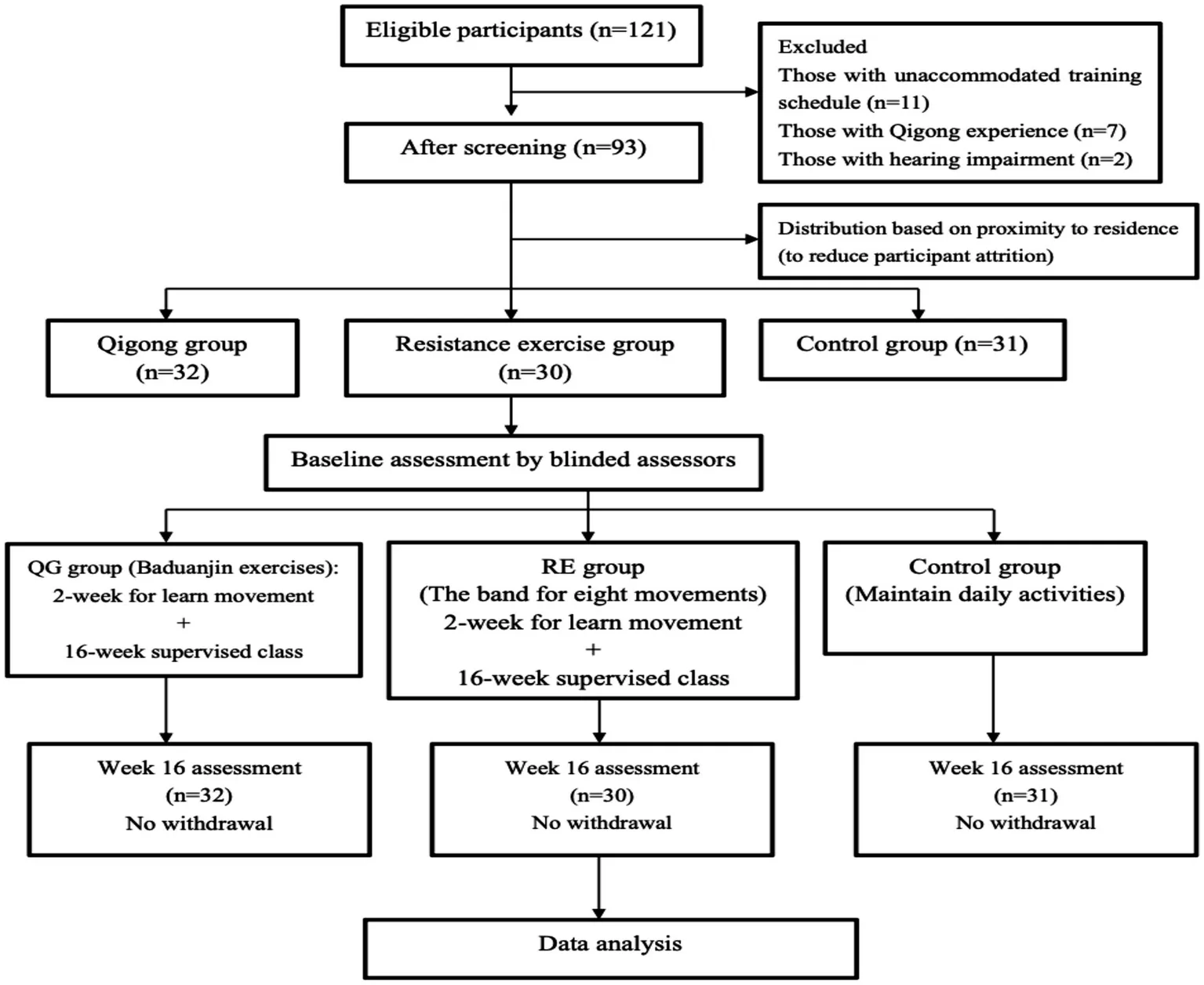
Flowchart diagram.

During the 16-week intervention, both experimental groups maintained a 100% attendance rate with no dropouts (Figure 2). There were no significant differences (*p* > 0.05) in training intensity (Baduanjin: 89 ± 8 b/min; Resistance Band: 88 ± 7 b/min) or daily physical activity (step count) between the two groups, and the RPE ratings (9–11) indicated that the exercise intensity was low to moderate (Table 3).

**Table 3:** Exercise intensity and daily activity amount during the experiment.

Time point	Training heart rate (THR) (b/min)		RPE (6–20)		Daily activity (steps)		
	QG (*N* = 32)	RE (*N* = 30)	QG (*N* = 32)	RE (*N* = 30)	QG	RE	CON
Week 1	83 ± 11	86 ± 10	9.7 ± 2.7	10.0 ± 3.0	3,769 ± 350	3,282 ± 279	3,190 ± 368
Week 5	82 ± 13	85 ± 13	9.8 ± 2.8	9.8 ± 2.8	3,324 ± 373	3,054 ± 258	3,328 ± 447
Week 9	81 ± 13	86 ± 14	9.8 ± 2.7	10.1 ± 3.1	2,991 ± 388	2,924 ± 252	3,138 ± 406
Week 16	82 ± 12	85 ± 13	9.8 ± 2.8	10.0 ± 3.1	3,021 ± 273	3,069 ± 168	3,108 ± 351

RE is the resistance exercise training group; QG is the qigong training group; CON is the control group. REP is rating of Perceived Exertion.

### 3.2 Improvement in balance ability

Static balance (CSST): after the intervention, the right-leg standing time in the eyes-closed single-leg stance test increased by 46% in the Baduanjin exercise group and 48% in the Resistance Band exercise group (*p* < 0.001), while the control group experienced a 31% decrease. There was no significant change in the left-leg standing time (*p* = 0.172). Between-group difference: for the right-leg performance, there was significant improvement (*p* = 0.003) in both groups, but no significant difference was found between the two training groups (p > 0.05).

Dynamic balance (TUG): a significant time × group interaction was observed (p < 0.001, η^2^ = 0.740). Test times decreased by 16% in the Baduanjin exercise group and by 10% in the Resistance Band exercise group, whereas they increased by 10% in the control group (Table 4). Although the Baduanjin exercise group showed greater improvement in dynamic balance than the Resistance Band exercise group, the difference between these two groups was not statistically significant (p > 0.05).

**Table 4:** ANOVA analysis of bodily function.

Indicator	Group	Before training	After training	ROC	Within-group comparison			Between-group comparison			Interaction effect			one-way ANCOVA			
		AVG ± STD	AVG ± STD		F	*p*	η^2^	F	*p*	η^2^	F	*p*	η^2^	M_adjust_ ± SE	F	*p*	η^2^
CSST-L (s)	RE	7.04 ± 4.15	9.35 ± 3.92	32.74%	1.898	0.172	0.021	1.602	0.207	0.034	16.16	<0.001	0.264	7.86 ± 0.42	108.42	<0.001	0.549
	QG	7.26 ± 3.88	7.87 ± 2.37	8.36%										9.48 ± 0.43			
	CON	7.45 ± 4.21	5.73 ± 4.01	−23.10%										5.60 ± 0.42			
CSST-R (s)	RE	5.49 ± 3.32	8.14 ± 3.08	48.34%	30.362	<0.001	0.254	6.217	0.003	0.123	48.182	<0.001	0.52	8.06 ± 0.3	122.92	<0.001	0.583
	QG	5.15 ± 2.61	7.53 ± 1.55	46.15%										7.67 ± 0.3			
	CON	5.45 ± 3	3.76 ± 2.71	−31.01%										3.70 ± 0.3			
TUG (s)	RE	10.23 ± 1.49	9.25 ± 1.34	−9.63%	51.803	<0.001	0.365	10.313	<0.001	0.186	127.992	<0.001	0.74	9.26 ± 0.1	316.864	<0.001	0.781
	QG	10.26 ± 1.38	8.58 ± 1.06	−16.35%										8.58 ± 0.1			
	CON	10.27 ± 1.55	11.35 ± 1.01	10.48%										11.33 ± 0.1			
BMI (kg/m^2^)	RE	23.16 ± 1.31	22.64 ± 1.21	−2.24%	105.017	0.000	0.539	0.34	0.713	0.008	34.702	<0.001	0.435	22.10 ± 0.07	1922.26	<0.001	0.956
	QG	23.12 ± 1.22	22.72 ± 1.21	−1.74%										22.01 ± 0.07			
	CON	23.13 ± 1.55	23.17 ± 1.47	0.17%										22.60 ± 0.06			
SBP (mmHg)	RE	138.8 ± 4.01	134.07 ± 3.95	−3.41%	87.66	0.000	0.493	12.165	<0.001	0.213	40.664	<0.001	0.475	133.81 ± 0.62	29.922	<0.001	0.252
	QG	138.12 ± 4.19	130.16 ± 3.97	−5.76%										130.22 ± 0.6			
	CON	137.94 ± 4.19	138.97 ± 3.72	0.75%										139.13 ± 0.6			
DBP (mmHg)	RE	88.57 ± 3.23	85.23 ± 3.07	−3.77%	65.202	0.000	0.42	11.007	<0.001	0.197	32.392	<0.001	0.419	85.23 ± 0.51	28.012	<0.001	0.239
	QG	88.56 ± 3.34	82.81 ± 3.31	−6.49%										82.81 ± 0.497			
	CON	88.55 ± 3.28	89.39 ± 3.23	0.95%										89.39 ± 0.5			
BST (cm, L)	RE	−1.8 ± 3.44	−0.3 ± 3.02	−83.33%	29.547	0.000	0.247	1.985	0.143	0.042	29.261	<0.001	0.394	−0.06 ± 0.35	196.18	<0.001	0.688
	QG	−1.5 ± 4.19	1.72 ± 3	−214.67%										1.73 ± 0.34			
	CON	−1.13 ± 4.06	−2.13 ± 4.23	88.50%										−2.38 ± 0.35			
BST (cm, R)	RE	−0.93 ± 3.52	0.5 ± 3.66	−153.76%	87.221	0.000	0.492	5.762	0.004	0.114	59.609	<0.001	0.57	0.54 ± 0.28	392.29	<0.001	0.815
	QG	−1.34 ± 3.69	2.66 ± 3.15	−298.51%										2.55 ± 0.27			
	CON	−2.1 ± 4.35	−2.68 ± 4.01	27.62%										−2.14 ± 0.28			
SRT (cm)	RE	6.47 ± 2.91	8.83 ± 2.89	36.41%	760.091	0.000	0.894	4.775	0.011	0.096	697.261	<0.001	0.939	8.72 ± 0.1	2398.98	<0.001	0.964
	QG	6.09 ± 3.14	9.86 ± 3.01	61.91%										10.12 ± 0.1			
	CON	6.53 ± 2.78	5.19 ± 2.77	−20.45%										5.03 ± 0.1			

RE is the resistance exercise training group; QG is the qigong training group; CON is the control group. ROC is rate of changes. CSST refers to the Closed-eyes Single-leg Standing Test, and TUG is the Timed Up and Go Test. SBP stands for systolic blood pressure, while DBP represents diastolic blood pressure. BST is the Back Scratch Test, and SRT is the Sit-and-Reach Test. The extremely high percentage gains observed in the Back Scratch Test are attributable to its unique scoring criteria.

### 3.3 Flexibility and blood pressure

There was no significant difference in BMI changes between the two groups (*p* = 0.713), indicating that the exercise load design did not significantly affect body composition. In terms of flexibility, the Baduanjin exercise group showed significantly greater improvements in both the right-shoulder Back Scratch Test (300% increase, *p* < 0.01) and the Sit-and-Reach Test (61.91% increase, *p* < 0.05) than the Resistance Band exercise group (150% increase, *p* = 0.068; 36.41% increase, *p* = 0.055) (Table 4). The extremely high percentage gains observed in the Back Scratch Test are attributable to its unique scoring criteria. Most participants had negative scores close to zero before the intervention. After training, their scores turned positive, so even a small absolute improvement produced a disproportionately large percentage increase. Additionally, the Baduanjin exercise group experienced more favorable improvements improvements in systolic blood pressure compared with the Resistance Band exercise group (*p* < 0.001), possibly due to the breathing regulation and sympathetic nervous system inhibition inherent in Baduanjin movements.

### 3.4 Cognitive function improvement

Both the Baduanjin exercise group and Resistance Band exercise group showed significantly greater improvements than the control group in the total MoCA score and its subdomains (naming, executive function, attention, orientation, and delayed recall) (*p* < 0.05), though no significant difference was found between the two training groups (Table 5). Notably, the Baduanjin exercise group demonstrated greater improvements in executive function (*p* = 0.037) and naming ability (*p* = 0.027) compared with the Resistance Band exercise group, suggesting that the integrated mind–body approach of Baduanjin may offer distinct advantages for specific cognitive domains.

**Table 5:** ANOVA analysis of cognitive function.

Cognitive domain	Group	Before training	After training	ROC	Within-group comparison			Between-group comparison			Interaction effect			one-way ANCOVA			
		AVG ± STD	AVG ± STD	Change	*F*	*p*	*η^2^*	*F*	*p*	*η^2^*	*F*	*p*	*η^2^*	M_adjust_ ± SE	*F*	*p*	*η^2^*
Executive function	RE	4.3 ± 0.7	4.93 ± 0.25	14.65%	14.209	0.000	0.136	0.938	0.395	0.02	4.013	0.021	0.082	4.94 ± 0.1	174.09	<0.001	0.662
	QG	4.5 ± 0.72	4.91 ± 0.39	9.11%										4.90 ± 0.1			
	CON	4.77 ± 0.72	4.77 ± 0.81	0.00%										4.78 ± 0.1			
Nominal function	RE	1.87 ± 0.51	2.5 ± 0.57	33.69%	21.787	0.000	0.195	8.383	0.000	0.157	8.617	0.000	0.161	2.55 ± 0.1	7.605	<0.001	0.079
	QG	2.28 ± 0.68	2.69 ± 0.47	17.98%										2.63 ± 0.1			
	CON	2.06 ± 0.57	2 ± 0.52	−2.91%										2.00 ± 0.1			
Linguistic ability	RE	2.67 ± 0.48	2.53 ± 0.51	−5.24%	0.583	0.447	0.006	0.376	0.688	0.008	0.948	0.391	0.021	2.50 ± 0.09	14.839	<0.001	0.143
	QG	2.56 ± 0.5	2.63 ± 0.49	2.73%										2.50 ± 0.09			
	CON	2.55 ± 0.51	2.48 ± 0.57	−2.75%										2.63 ± 0.09			
Attention span	RE	4.97 ± 0.81	5.43 ± 0.77	9.26%	4.495	0.037	0.048	5.753	0.004	0.113	10.668	0.000	0.192	5.45 ± 0.14	11.906	<0.001	0.118
	QG	5.09 ± 0.78	5.66 ± 0.65	11.20%										5.63 ± 0.14			
	CON	5.03 ± 0.66	4.61 ± 1.09	−8.35%										4.61 ± 0.14			
Orientation	RE	5.07 ± 0.83	5.2 ± 0.89	2.56%	0.123	0.727	0.001	5.126	0.008	0.102	8.734	0.000	0.163	5.2 ± 0.2	0.206	0.651	0.002
	QG	4.94 ± 0.8	5.47 ± 0.76	0.1073										5.47 ± 0.2			
	CON	5.1 ± 0.75	4.29 ± 1.44	−0.1588										4.29 ± 0.2			
Abstract reasoning	RE	2.13 ± 0.68	1.83 ± 0.46	−0.1408	1.94	0.167	0.021	1.008	0.369	0.022	8.101	0.001	0.153	1.81 ± 0.07	5.0.494	<0.005	0.058
	QG	1.75 ± 0.44	2.03 ± 0.18	0.16										2.07 ± 0.07			
	CON	2.16 ± 0.64	1.9 ± 0.54	−0.1204										1.87 ± 0.07			
Delayed recall	RE	2.4 ± 1	4.37 ± 0.67	0.8208	83.246	0.000	0.481	3.463	0.036	0.071	20.077	0.000	0.309	4.45 ± 0.2	8.395	<0.001	0.086
	QG	2.72 ± 1.14	4.37 ± 0.98	0.6066										4.37 ± 0.18			
	CON	2.97 ± 1.14	3 ± 1.41	0.0101										2.92 ± 0.18			

RE is the resistance exercise training group; QG is the qigong training group; CON is the control group. ROC is rate of changes.

In terms of the correlation between balance ability and cognitive function, dynamic balance, as assessed by the TUG, was moderately negatively correlated with the total MoCA score (r = −0.80, *p* < 0.01), while static balance, measured by the CSST, showed a slightly weaker negative correlation (r = −0.31, *p* < 0.01). These findings support the hypothesis that better balance is associated with superior cognitive function (Table 6). Further analysis revealed a significant negative correlation between executive function and TUG (r = −0.28, *p* < 0.05), indicating that dynamic balance demands higher-order cognitive processes. Additionally, attention was moderately negatively correlated with both TUG (r = −0.35) and CSST (r = −0.33) (*p* < 0.01), reflecting the cognitive-motor dual-task coordination mechanism.

**Table 6:** Correlation analysis between balance ability and cognitive function.

Cognitive index	CSST-L (s)	CSST-R(s)	TUG (s)
MoCA	0.42**	0.70**	−0.80**
Executive function	0.14	0.17	−0.19
Nominal Function	0.09	0.31**	−0.32**
Attention Span	0.30**	0.35**	−0.39**
Linguistic Ability	−0.044	−0.084	0.01
Abstract Reasoning	0.02	0.12	−0.23*
Delayed Recall	0.22*	0.43**	−0.45**
Orientation	0.20	0.30**	−0.34**

CSST refers to the Closed-eyes Single-leg Standing Test; TUG is the Timed Up and Go Test; MoCA is the Montreal Cognitive Assessment. ** indicates that the correlation is significant at the 0.01 level (two-tailed); * indicates that the correlation is significant at the 0.05 level (two-tailed).

Analysis of Covariance (ANCOVA) was used to control for potential confounding factors, including age and gender, thereby minimizing their possible influence on the results. Additionally, baseline differences were controlled to ensure the reliability of the findings. Baseline comparisons revealed differences in cognitive function scores across the groups. Specifically, significant differences were observed in multiple cognitive domains: executive function (*p* = 0.037), nominal function (*p* = 0.027), and abstract reasoning (*p* = 0.011). No significant differences were found in attention span (*p* = 0.801), linguistic ability (*p* = 0.600), delayed recall (*p* = 0.136), or orientation (*p* = 0.699) (see Table 1). These analyses helped to enhance the accuracy and comparability of the study results.

For balance ability and physical function, the time × group interaction was significant for the Closed-eyes Single-leg Standing Test (CSST) (*p* < 0.001, η^2^ = 0.520) and the Timed Up and Go (TUG) test (*p* < 0.001, η^2^ = 0.740; see Table 4). Post-hoc analyses indicated that after the intervention, both the Baduanjin and Resistance Band exercise groups improved their balance, whereas the control group showed declines to varying degrees. Within-group comparisons revealed that changes in the right-leg CSST (CSST-R) were significant (*p* < 0.001), whereas those in the left-leg CSST (CSST-L) were not (*p* = 0.172). Between-group comparisons showed a significant difference for CSST-R (*p* = 0.003), but not for CSST-L (*p* > 0.05). The Baduanjin and Resistance Band exercise groups improved right-leg balance by 46 and 48%, respectively, while the control group declined by 31%. Further post-hoc analysis indicated that the differences between the two training groups in the balance test were not significant (*p* > 0.05). For the TUG test, the time × group interaction was significant (*p* < 0.001, η^2^ = 0.740), and between-group differences were also significant (*p* < 0.001). Post-hoc analyses showed that the Resistance Band and Baduanjin groups reduced their test times by 10 and 16%, respectively, whereas the control group’s time increased by 10%. Further between-group comparisons indicated that the improvements in TUG performance did not differ significantly between the two training groups (p > 0.05).

After 16 weeks of training, both Resistance Band and Baduanjin exercises produced positive effects on blood pressure and flexibility in older adults, although the effects varied across different indicators (see Table 4). Regarding BMI, both training groups improved by approximately 2%, but there was no significant difference compared with the control group (*F* = 0.340, *p* = 0.713, η^2^ = 0.008). For the flexibility tests, there was no significant inter-group difference in the left-shoulder Back Scratch Test (*p* > 0.05), but the right-shoulder Back Scratch Test showed a significant difference among groups (*F* = 5.762, *p* = 0.004, η^2^ = 0.114). Compared with a 28% decrease observed in the control group, the Resistance Band group improved by 150% (*p* = 0.068) and the Baduanjin group by 300% (*p* < 0.01). In the Sit-and-Reach Test (SRT), a significant between-group difference was found (*F* = 4.775, *p* = 0.011, η^2^ = 0.096): the Baduanjin group improved by 61.91%, the Resistance Band group by 36.41%, while the control group declined by 20.45%. Further analysis revealed that the Resistance Band exercises group showed a trend toward improvement (*p* = 0.055), whereas the Baduanjin group significantly enhanced flexibility (*p* < 0.05). Overall, Baduanjin exercises appeared to be slightly more effective than Resistance Band exercises in improving flexibility. In terms of blood pressure, both training methods were significantly more effective than the control condition, with significant between-group differences in systolic blood pressure (*p* < 0.001); moreover, the Baduanjin group showed greater improvement than the Resistance Band exercises group.

Regarding cognitive performance, both the Baduanjin and Resistance Band groups had positive effects on older adults compared with the control group, which exhibited a significant decline. Between-group comparisons revealed that both training groups significantly outperformed the control group on the MoCA subtests for Nominal Function (Visuospatial Skills), Executive Function, Attention Span, Orientation, and Delayed Recall (*p* < 0.05). However, no significant improvements were observed across the three groups in Linguistic Ability or Abstract Reasoning (see Table 5).

Based on the results presented above, both Baduanjin and Resistance Band exercises had positive effects on balance, flexibility, blood pressure and other aspects in older adults, and each training group also showed varying degrees of improvement in cognitive function. Further analysis revealed a moderate to high positive correlation between cognitive function and balance ability in older adults—that is, higher cognitive level was associated with better balance (see Table 6). Specifically, the nominal function (visuospatial skills) showed a low positive correlation with dynamic balance ability (TUG: r = −0.32, *p* < 0.01), and static balance (CSST: r = −0.31, *p* < 0.01). In addition, abstract reasoning was also lowly positively correlated with dynamic balance (TUG: r = −0.23, *p* < 0.05). Attention Span was moderately positively correlated with dynamic balance ability (*p* < 0.01), while both Delayed Recall and Orientation showed low to moderate positive correlations with dynamic balance. These findings further confirm the close relationship between cognitive function and balance ability, suggesting that enhancing cognitive function may help improve balance in older adults (48).

## 4 Discussion

Prior to interpreting the core intervention results, this paper clarifies the handling of baseline cognitive disparities caused by the non-random grouping design. Participants were allocated based on residential proximity rather than randomization, resulting in significantly higher baseline MoCA total scores in the control group compared to the two exercise arms (Table 1). Analysis of Covariance (ANCOVA) was applied for all cognitive outcome analyses, with baseline MoCA scores set as covariates to adjust post-intervention marginal means; all between-group efficacy comparisons presented below have eliminated confounding bias from initial cognitive differences.

This study investigated the effects of a 16-week intervention of Baduanjin and Resistance Band exercises on the balance and cognitive function in community-dwelling older adults. The key findings are threefold. First, both interventions significantly improved balance and cognitive function relative to the control group, with comparable efficacy in static balance (CSST), dynamic balance (TUG) and global cognitive function (MoCA), as no significant differences were observed between the two training groups on these measures. Second, domain-specific differences emerged between the two interventions: Baduanjin demonstrated significantly greater improvements in flexibility (BST and SRT), systolic blood pressure, and specific cognitive subdomains including executive function and naming ability. Third, a significant negative correlation was observed between dynamic balance (TUG) and global cognitive function (MoCA total score, r = −0.80), further supporting the close relationship between balance and cognition. Notably, both interventions were delivered at low-to-moderate intensity with comparable exercise loads and daily activity levels, and the 16-week program achieved 100% attendance with no dropouts, confirming the feasibility and safety of these community-based exercise protocols. These findings collectively suggest that both Baduanjin and Resistance Band training are effective and feasible interventions for promoting balance and cognitive health in older adults, with each modality offering distinct benefits across different outcome domains.

### 4.1 Domain-specific effects of Baduanjin and resistance band training on balance

Both Baduanjin and Resistance Band exercises significantly enhanced balance ability in older adults relative to the control group, with no significant between-group differences in either static balance (CSST) or dynamic balance (TUG) (see Table 4). Regarding the first research question, these findings indicate that the two modalities produce comparable improvements in balance over the 16-week intervention period, despite their fundamentally different movement characteristics.

The comparable balance improvements may be attributed to distinct yet convergent mechanisms. Baduanjin enhances balance through frequent shifts in the center of gravity, variations in lower limb postures, and coordinated movements that improve neuromuscular coordination and proprioceptive control (8, 9, 29). These vestibular and neural adaptive changes could not be objectively quantified in this study. Resistance Band training, in contrast, primarily improves balance through enhanced muscle strength and joint stability, as external resistance challenges the lower limb musculature responsible for postural control (18). This finding aligns with previous evidence that both mind–body exercises and resistance-based training can effectively improve balance in older adults, though through different pathways—Baduanjin through sensory-motor integration and Resistance Band through muscular conditioning.

Notably, beyond balance measures, domain-specific differences did emerge. Baduanjin demonstrated significantly greater improvements in flexibility—as measured by both the Sit-and-Reach Test and the right-shoulder Back Scratch Test—and in systolic blood pressure compared with the Resistance Band group (*p* < 0.05; see Table 4). These differential effects are consistent with the distinct movement characteristics of Baduanjin, which incorporates extensive stretching, rotational movements, and controlled breathing that promote tissue elasticity and parasympathetic activation (33, 38). In contrast, Resistance Band exercises involve a relatively limited range of motion due to the constraints of the elastic resistance. The superior blood pressure improvement in the Baduanjin group is consistent with prior evidence that mind–body exercises emphasizing relaxation and respiratory regulation can reduce sympathetic nervous system excitability and improve vascular function (38, 50).

### 4.2 Domain-specific effects of Baduanjin and resistance band training on cognitive function

Both interventions produced significant improvements in cognitive function relative to the control group, with no significant difference between the two training groups in MoCA total score. However, at the subdomain level, the Baduanjin group demonstrated significantly greater improvements in Executive Function (*p* = 0.037) and Nominal Function (*p* = 0.027) compared with the Resistance Band group (see Table 5). These domain-specific differences suggest that the integrated mind–body approach of Baduanjin may confer distinct advantages for certain cognitive domains, particularly those involving visuospatial processing and executive control.

Baduanjin exercises emphasize mental regulation along with coordinated movements, which can better activate attention and executive function in older adults (39, 40). In contrast, although Resistance Band exercises can also improve cognitive function, their effects are mainly manifested through enhancement of muscle strength, with relatively limited direct stimulation of cognitive function (41). Additionally, the movement design of Baduanjin exercises combines breathing regulation with mental concentration, which is speculated to boost neuroplasticity and cerebral blood circulation based on existing neuroimaging evidence (20, 42). This mind–body training approach may be a key factor contributing to the domain-specific cognitive improvements observed here, though such neural changes were not directly measured in the current trial. Notably, the control group exhibited a marked decline in cognitive function after the 16-week period, further demonstrating the protective effect of exercise interventions against cognitive decline in older adults (43).

### 4.3 Correlation between balance ability and cognitive function

**Figure 3:**
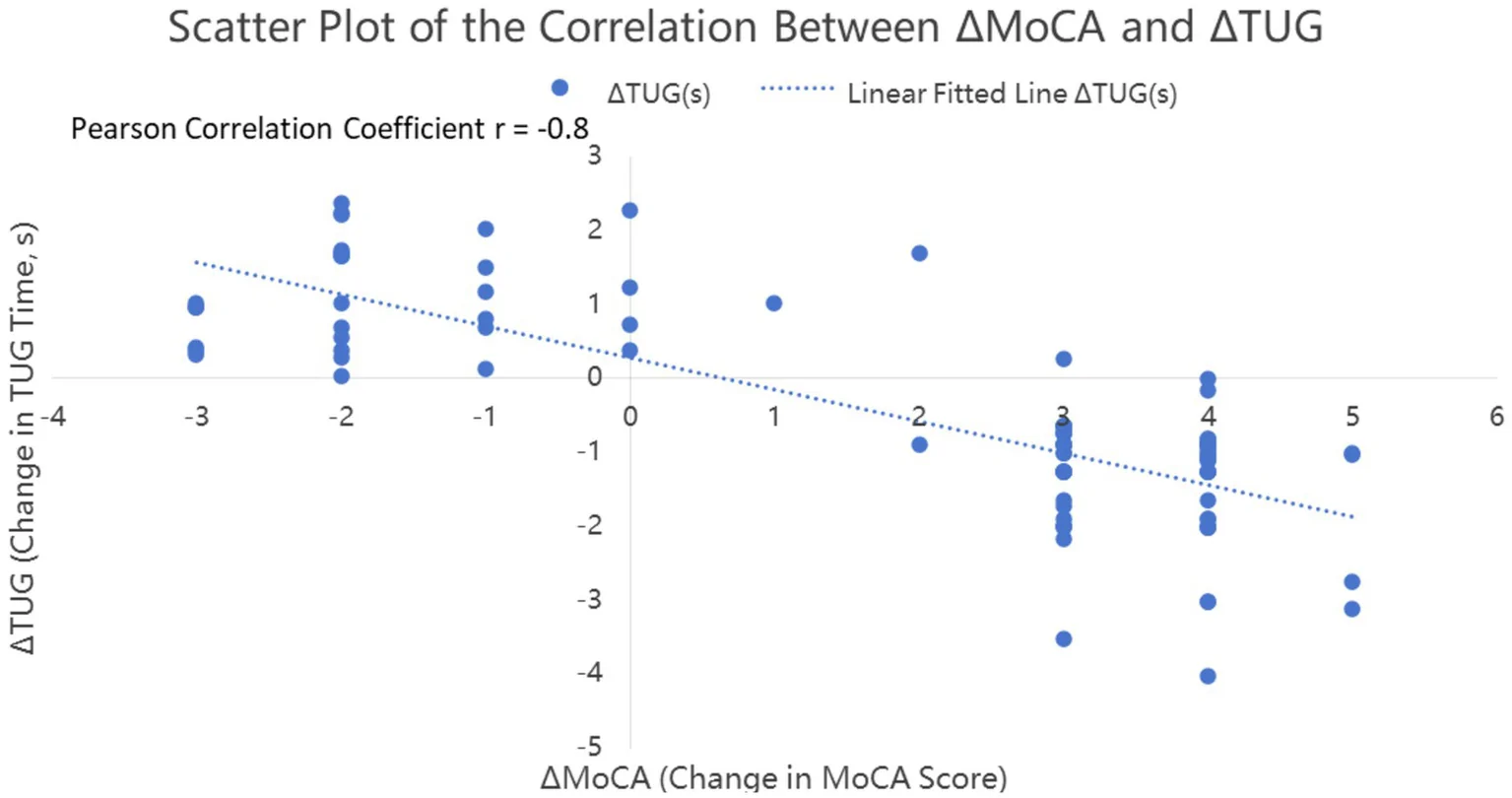
Scatter plot of the correlation between *ΔMoCA* and *ΔTUG*. TUG is the Timed Up and Go Test; MoCA is the Montreal Cognitive Assessment.

A significant positive correlation was observed between balance ability and cognitive function. Specifically, dynamic balance ability (TUG & Timed Up and Go Test) was moderately to strongly correlated with several cognitive dimensions, including global cognition (MoCA total score, r = −0.80, *p* < 0.01) attention (r = −0.32) and delayed recall (r = −0.45) (see Table 6). This finding supports the hypothesis that better balance is associated with superior cognitive function and is consistently with previous research demonstrating that gait speed and dynamic balance are closely related to cognitive function (44, 51, 52). Potential outliers in the correlation analysis between MoCA total score and TUG time were carefully inspected, and supplementary scatter plots were provided to verify the correlation results (Figure 3).

Notably, the pattern of correlations varied across cognitive domains: executive function exhibited a weaker correlation with TUG (r = −0.19) compared with global cognition or attention. This domain-specific pattern is broadly consistent with previous findings that global cognitive measures may capture broader cognitive-motor integration than isolated executive function tests (51, 52). However, this study did not find a significant correlation between executive function and balance ability, which diverges from the findings of Jovanović et al.'s (45) meta-analysis. These discrepancies suggest that the relationship between specific cognitive domains and balance may be more nuanced than previously assumed, potentially influenced by the specific tests used and population characteristics (Table 2).

Regarding the second research question—whether these correlations are moderated by exercise type—the present study did not formally examine this due to limited statistical power for stratified correlation analyses. Consequently, whether the balance-cognition relationship differs between mind–body and resistance exercise modalities remains an open question that warrants further investigation with larger samples (49).

### 4.4 Limitations and future directions

This study has several limitations. First, participants were assigned to groups based on residential proximity rather than through random allocation, which may have introduced selection bias and limits causal attribution. To ensure a high participant retention rate during the trial, we did not implement random grouping, which may have exerted certain impacts on the statistical results. Future research should strive to balance adequate sample size and rigorous random allocation design. Second, although the 16-week intervention period exceeded the typical duration of short-term studies (8), long-term effects remain unverified. Third, participants were predominantly healthy community-dwelling older adults, limiting generalizability to populations with chronic conditions (17). Fourth, the study did not collect biological biomarkers (e.g., peripheral BDNF) or neuroimaging scans (e.g., fMRI for gray matter quantification) to directly validate the proposed neural and vestibular mechanistic pathways linking exercise to balance and cognitive gains (11). All interpretations regarding neuroplasticity, cerebral perfusion and vestibular compensation remain speculative and are only supported by prior literature rather than objective measurements from this cohort. Follow-up trials integrating neuroimaging and blood biomarker testing are needed to verify these physiological pathways. Fifth, the sample size was insufficient for stratified correlation analyses to examine whether exercise type moderates the balance-cognition relationship. Future research should employ randomized designs, incorporate biomarker and neuroimaging measures, extend intervention and follow-up durations, and include larger samples to enable subgroup analyses. Apart from the above constraints, two additional methodological confounders should be acknowledged. First, regular on-site training supervision may induce the Hawthorne effect, whereby participants alter their daily behaviors or test performance due to being continuously observed. Second, all participants live within the same community and may communicate training content and experience with one another, leading to cross-group contamination that could blur true intergroup differences. Future community-based trials should adopt independent residential blocks for different groups and reduce frequent on-site monitoring to mitigate these biases.

## 5 Conclusion

This study demonstrates that both Baduanjin and Resistance Band exercises are effective in improving balance and cognitive function in older adults, with each modality conferring distinct benefits across specific outcome domains. Baduanjin demonstrated superior improvements in flexibility, blood pressure, and specific cognitive subdomains including executive function and visuospatial/naming function, while Resistance Band training produced comparable improvements in balance and global cognitive function. The significant correlation between balance and cognitive function further supports the close relationship between physical and cognitive health in aging populations. These findings suggest that both interventions are safe, feasible and accessible options for community-based exercise prescription, with the choice between them depending on individual health priorities. Future research incorporating biomarker and neuroimaging measures is needed to elucidate the distinct mechanisms underlying the cognitive benefits of these two exercise modalities.

## Data Availability

The raw data supporting the conclusions of this article will be made available by the authors, without undue reservation.
